# Gasdermins in Innate Host Defense Against *Entamoeba histolytica* and Other Protozoan Parasites

**DOI:** 10.3389/fimmu.2022.900553

**Published:** 2022-06-20

**Authors:** Shanshan Wang, France Moreau, Kris Chadee

**Affiliations:** Department of Microbiology, Immunology and Infectious Diseases, University of Calgary, Calgary, AB, Canada

**Keywords:** gasdermin, parasite, macrophage, *Entamoba histolytica*, innate immunity

## Abstract

Gasdermins (GSDMs) are a group of proteins that are cleaved by inflammatory caspases to induce pore formation in the plasma membrane to cause membrane permeabilization and lytic cell death or pyroptosis. All GSDMs share a conserved structure, containing a cytotoxic N-terminal (NT) pore-forming domain and a C-terminal (CT) repressor domain. *Entamoeba histolytica* (*Eh*) in contact with macrophages, triggers outside-in signaling to activate inflammatory caspase-4/1 *via* the noncanonical and canonical pathway to promote cleavage of gasdermin D (GSDMD). Cleavage of GSDMD removes the auto-inhibition that masks the active pore-forming NT domain in the full-length protein by interactions with GSDM-CT. The cleaved NT-GSDMD monomers then oligomerize to form pores in the plasma membrane to facilitate the release of IL-1β and IL-18 with a measured amount of pyroptosis. Pyroptosis is an effective way to counteract intracellular parasites, which exploit replicative niche to avoid killing. To date, most GSDMs have been verified to perform pore-forming activity and GSDMD-induced pyroptosis is rapidly emerging as a mechanism of anti-microbial host defence. Here, we review our comprehensive and current knowledge on the expression, activation, biological functions, and regulation of GSDMD cleavage with emphases on physiological scenario and related dysfunctions of each GSDM member as executioner of cell death, cytokine secretion and inflammation against *Eh* and other protozoan parasitic infections.

## Introduction

The gastrointestinal tract encounters innumerable luminal insults, including those caused by noxious substances, commensal bacteria, and/or pathogens. The enteric protozoan parasite *Entamoeba histolytica* (*Eh*) is the causative agent of intestinal amebiasis, which manifests as amebic colitis and amebic liver abscess (1, 2) and is named for its ability to lyse host tissues. Of approximately 10 individuals that are infected, one will develop symptoms such as diarrhea and abdominal pain (3). On rare circumstances, *Eh* penetrates the intestinal mucosa and cause necrosis that extend to the submucosa and muscularis, causing “flask-shaped ulcer” (3, 4) and enter the bloodstream and disseminate to other extraintestinal sites, most commonly the liver, lung and brain. Collectively, there are approximately 100 million annual cases of amebic dysentery, colitis and liver abscess resulting 11,300 deaths in 2013 (5).

**Figure 1:**
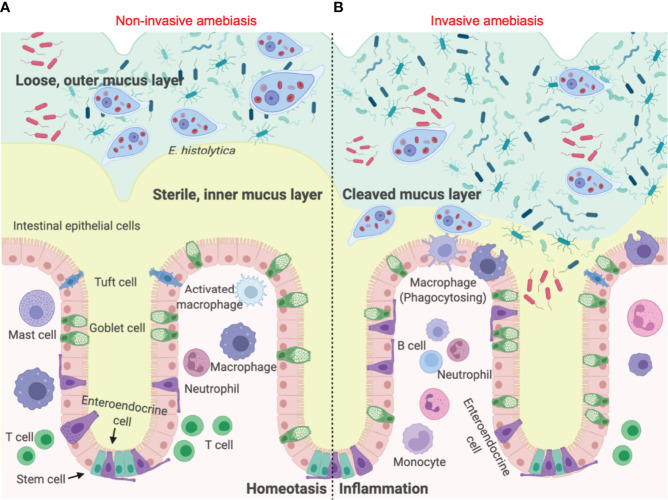
*Entamoeba histolytica* breaches the mucus layer and results in invasive amebiasis. **(A)** When *Eh* infects a healthy individual it colonizes the looser outer mucus layer by binding Gal/GalNAc mucin glycans to the parasite Gal-lectin adhesin (10). Here the parasite feeds on bacteria, cellular debris and sugar from mucus to satisfy its energetic needs, establishing asymptomatic infections. **(B)** In invasive amebiasis *Eh* disrupts innate host defences by cleaving mucin glycans with parasite glycosidases followed by the degradation of MUC2 mucin in its C-terminal domain *via Eh* cysteine proteases to dissolve the protective colonic mucus gel (11). This allows *Eh* to come in direct contact with and disrupts intestinal epithelial cells where the parasite comes in direct contact with lamina propria macrophages that express high amounts of NLRP3 inflammasomes evoking a prompt pro-inflammatory response dominated by TNF-α, IL-1β and IL-8.

The first line of innate host defence in the gastrointestinal tract is the mucus layer produced by goblet cells (6). In the colon, the epithelial barrier consists of three layers: the single layer of intestinal epithelial cells (IECs) that separates the underlying lamina propria, the mucus bilayer and indigenous microbiota that reside in/on the mucus layer (7). The colonic mucus layer contains two components: a firm sterile inner mucus layer adherent on the apical surface of IECs (8), and a superficial loose outer mucus layer that is heavily colonized by commensal microorganisms (9) (**Figure 1A**). Colonic MUC2 mucins are the structural component of the mucus gel that are rich in galactose (Gal) and N-acetyl galactosamine (GalNAc) (11). *Eh* colonizes the gut by binding of the surface Gal/GalNAc adherence lectin (Gal-lectin) adhesin to mucin Gal and GalNAc glycans. Here it also uses the Gal-lectin adhesin to bind bacteria and host cells that it feeds upon (12–15). In disease pathogenesis, *Eh* cysteine proteases degrades peptides in the poor glycosylated regions of the MUC2 C-terminus (16) to abrogate mucus protective functions allowing the parasite to penetrate through the mucus layer (17) (**Figure 1B**). In addition to mucin degradation, *Eh* also evokes hypersecretion (exocytosis) of mucus by engaging *Eh* cysteine protease-5 (*Eh*CP-A5) RGD motif (arginine-glycine-aspartate) to α_v_β_3_ integrin on goblet cells (18) as a means not only to defend against the parasite but also to deplete intracellular mucin stores.

**Figure 2:**
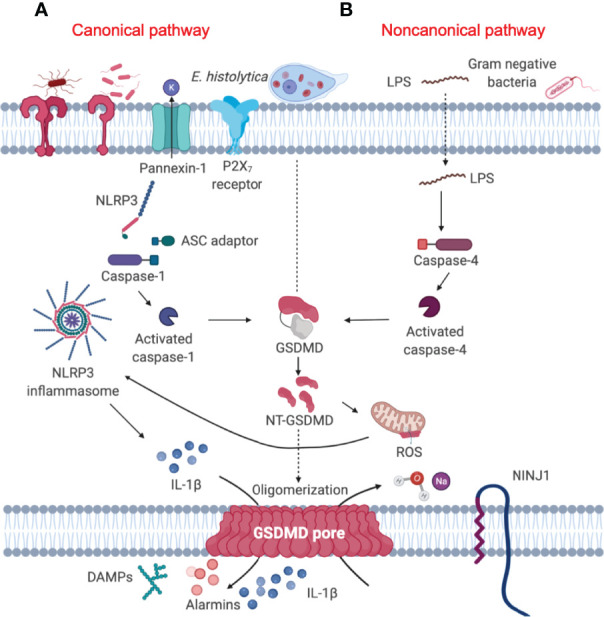
Gasdermin D-mediated pyroptosis is regulated by both canonical and noncanonical signaling pathway. **(A)** Canonical inflammasome pathway (left) constitute a Nod-like receptor (NLRP3), the adaptor protein ASC and pro-caspase-1, which converts pro-IL-1β into its active form. Activated caspase-1 cleaves GSDMD to unleash the NT-GSDMD, which mediates bioactive IL-1β release and ultimately causes cell pyroptosis (19). **(B)** Cytosolic LPS released from Gram-negative bacteria (20) and the extracellular protozoan *Eh* can trigger the activation of caspase-4 (21), leading to activation of the noncanonical signaling pathway (right). Activated caspase-4 in turn, cleaves GSDMD in a similar fashion as canonical caspase-1 to mediate the secretion of mature pro-inflammatory cytokines (22). NINJ1 is a cell-surface protein that contains two transmembrane domains and is a mediator of plasma membrane rupture that can release pro-inflammatory cytokines (23).

When the mucus barrier is degraded and/or depleted by mucus hypersecretion that exceeds MUC2 biosynthesis, *Eh* comes in contact with and disrupts the surface epithelium. This allows *Eh* to interact with resident macrophages in the lamina propria to elicit a robust pro-inflammatory response through activation of the nucleotide-binding oligomerization domain (NOD)-like receptor-pyrin containing 3 (NLRP3) inflammasome, leading to interleukin (IL)-1β and IL-18 release (10) (**Figure 2A**). The direct sensing of *Eh via* Gal-lectin induces NLRP3 inflammasome activation, driving the processing and release of IL-1β and an aggressive inflammatory response that are aimed at eradicating *Eh* and recruiting immune cells to the site of infection (10). The canonical NLRP3 inflammasomes are the cytosolic sensor that target pathogens and various cellular stresses that form multimeric high molecular complexes to mediate recruitment and activation of caspase-1 (24). Until recently, most work on the NLRP3 inflammasome has been focused on the functions of caspase-1 (25–29). However, recent studies using *Casp1^–/–^* mice that have a mutation in *Casp11*, led to the discovery of the noncanonical inflammasome pathway regulated by caspase-11 and identification of a lytic cell death pathway induced by caspase-11 independent of the canonical NLRP3 inflammasome complex (30). Most gram-negative bacteria such as *Escherichia coli*, *Citrobacter rodentium* and *Vibrio cholerae*, activate caspase-11/4-mediated signaling pathway in mouse/human macrophages resulting in maturation and release of IL-1β/IL-18 and pyroptosis (30) (**Figure 2B**). However, the precise role of caspase-11/4 in macrophage inflammasome signaling and their mechanism of activation against parasitic infections remain to be clarified.

**Figure 3:**
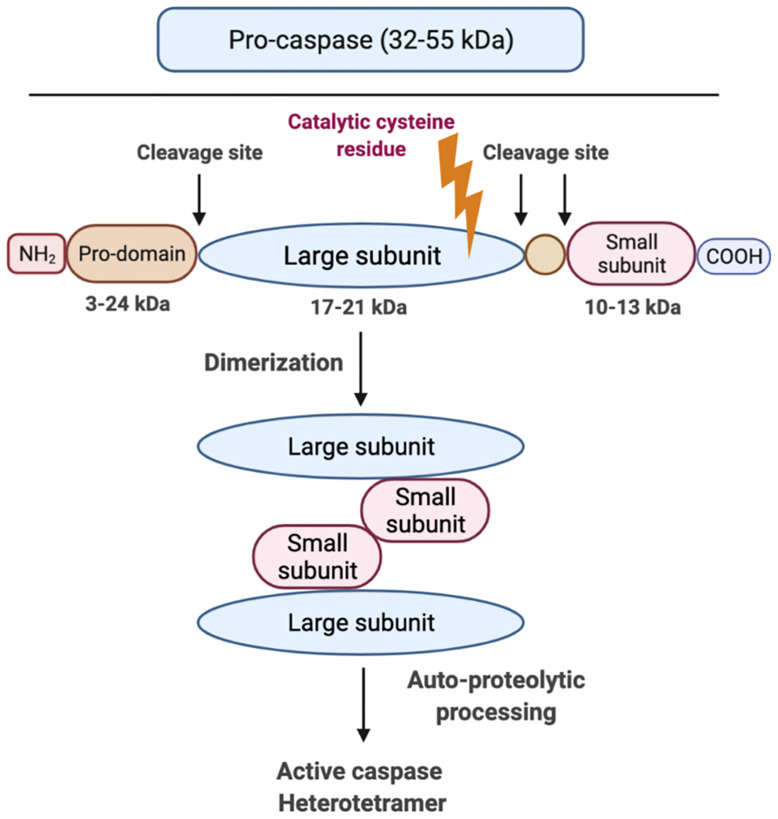
Graphic representation of caspase structure. Caspases share a common structure, consisting of a 3-24 kDa NT pro-domain, a 17-21 kDa central large domain and a 10-13 kDa small CT subunit domain. All caspases consist of an active cysteine residue (bold lightning) in the large domain that specifically cleaves after the aspartate residue in substrates. The activation of inflammatory caspases usually requires two steps: step 1 is dimerization that is induced by proximity and step 2 is auto-processing within the catalytic domain of each caspase monomer.

Although caspases have different effector functions, all caspases share a common structure: caspases constitute an N-terminal (NT) pro-domain, a central large domain containing an active cysteine residue and a small C-terminal (CT) subunit domain (31). The pro-domains of caspases contain caspase activation and recruitment domains (CARDs) or death effector domains (DEDs). A growing body of literature suggests that the activation of inflammatory caspase requires at least two steps: the catalytic component of two pro-caspases need to undergo dimerization, which is made up of two large and two small subunits (**Figure 3**). Following this, autocleavage is triggered within the dimerized full protease (32). Auto-proteolytic processing of pro-caspase occurs when the protein is cleaved between the pro-domain and the large subunit as well as between the large subunit and the small subunit, which allows both large/small subunits to re-assemble as an active heterotetramer (31) (**Figure 3**). Caspases need to be tightly regulated as their hyperactivation can promote auto-inflammatory diseases, while insufficient activation of caspases can lead to susceptibility to infection and sepsis (33). It is still unclear how each specific caspase works, if there is any redundancy or interaction among them and how caspase functions are fine-tuned.

Upon sensing multiple pathogen and host-derived cellular insults *via* pathogen and damage-associated molecular patterns (PAMPs and DAMPs, respectively), canonical NLRP3 sensor triggers oligomerization of the adaptor protein apoptosis-associated speck like protein containing a CARD (ASC) and the effector protein caspase-1 through homotypic interactions, leading to the formation of the multimeric inflammasome complex to regulate cytokine maturation and pyroptosis. Yet, the accurate mechanism and interaction governing the crosstalk between canonical NLRP3 and noncanonical caspase-4/5/11 inflammasomes in response to parasites require further elucidation. It has been suggested that caspase-4 and caspase-5 may have originated from caspase-11 (34) as caspase-11 share approximately 60% amino acid identity to caspase-4 (35). Hence, there is a degree of crosstalk between the inflammatory caspases that determine the fate of the cell, and manage the outcome of host response to infections. Macrophages deficient in caspase-4 have decreased caspase-1 activation and reduced IL-1β secretion (22) when stimulated with ATP and monosodium urate crystals (MSU) (36), suggesting caspase-4 functions similarly to caspase-11, as it might regulate caspase-1 activation. The distinct role of caspase-4 is also implicated in dengue virus infection where it was discovered to be critical in regulating caspase-1 activity and subsequent IL-1β secretion (37). By serendipitous discovery, caspase-11 and the human ortholog caspase-4 are shown to directly bind lipopolysaccharide (LPS) *via* their CARD domains that undergo oligomerization and activation (38). Though caspases are known to require the assembly of receptor complex for their activation, caspase-11 and caspase-4 appear to occur without the formation of a specific receptor complex. Cytosolic LPS released from gram-negative bacteria is detected by murine caspase-11 or human caspase-4 and 5, leading to activation of the noncanonical inflammasome (**Figure 2B**). These activated inflammatory caspases in turn, cleave GSDMD to cause cell pyroptosis. Similar to macrophages, caspase-4/1 initiate pyroptotic cell death in epithelial cells infected with *S. typhimurium* to restrict bacterial growth (39). These studies show that caspase-4 activation is important for inflammatory signaling processes, displaying diverse functions, however, extensive studies are required to decipher caspase-4 and caspase-11 functions in inflammatory response evoked by protozoan parasites.

## The Gasdermin Family

The gasdermins (GSDMs) are a family of recently identified intracellular proteins that elicit pyroptosis. The human genome encodes six paralogous members of GSDM family proteins: GSDMA, GSDMB, GSDMC, GSDMD, GSDME/DFNA5 and DFNB59 (also known as pejvakin) (40), and ten in mouse, including GSDMA1, GSDMA2, GSDMA3, GSDMC, GSDMC2, GSDMC3, GSDMC4, GSDMD, GSDME, DFNB59 (40, 41). The name gasdermin comes from the expression in the gastrointestinal tract and epithelium of the skin. All members of the GSDM family excluding DFNB59 are conserved and comprised of highly conserved NT and CT fragments that are separated by a central flexible and viable linker that harbour pore-forming activity (42). Upon cleavage by caspases or granzymes, the NT fragment of GSDMs binds to acidic phospholipids in the inner leaflet of cell membranes to form GSDM pores (42–45).

### GSDMA

**Figure 4:**
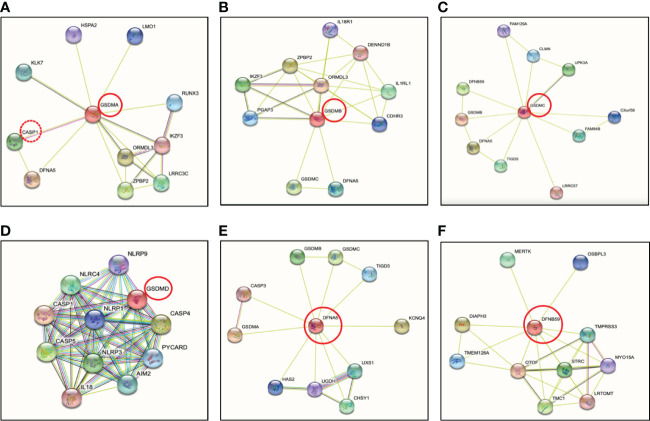
Protein-protein interactions of the human gasdermin family members by STRING analysis. **(A)** GSDMA is cleaved by caspase-1 with predicted interaction between GSDMA and DFNA5. GSDMA is co-expressed with KLK7, IKZF3 and ORMDL3. **(B)** GSDMB is co-expressed with ORMDL3 as GSDMA. ORMDL3 is ORM1-like protein 3, a negative regulator of sphingolipid synthesis, which may indirectly regulate endoplasmic reticulum-mediated Ca^2+^ signaling. **(C)** The co-expression of GSDMC is UPK3A or uroplakin-3a, is a component of the asymmetric unit membrane (AUM). UPK3A is a highly specialized biomembrane elaborated by terminally differentiated urothelial cells, that play an important role in preventing bacterial adherence. **(D)** GSDMD is essential effector of NLRP3 inflammasome-dependent caspase-1 activation and IL-1β/IL-18 secretion in response to noncanonical activators, including UVB radiation, cholera enterotoxin subunit B and cytosolic LPS. **(E)** GSDME or nonsyndromic hearing impairment protein 5 (DFNA5) plays a role in the TP53-regulated cellular response to DNA damage probably by cooperating with TP53, which is known to activate the transcription of numerous tumor suppressors and activators of apoptosis. In addition to pyroptotic activity, GSDME interacts with caspase-3/7 to trigger apoptosis by targeting mitochondria, but is more strongly implicated in tumor suppression. **(F)** Protein-protein interaction analysis shows no direct connection between DFNB59 and other GSDMs. Otoferlin (OTOF) is a key Ca^2+^ sensor involved in Ca^2+^ dependent SNARE-mediated exocytosis. Possible interaction between DFNB59 and OTOF suggests DFNB59 might be involved in vesicle transportation but requires further confirmation.

The human genome encodes a single GSDMA, whereas the mouse genome encodes three paralogs known as GSDMA1, GSDMA2 and GSDMA3 (46–48). Mouse GSDMA1 was the first cloned member of the GSDM family protein and was found exclusively in the upper gastrointestinal tract (46). Human GSDMA is expressed in skin and the gastrointestinal tract, including the epidermis, hair follicles, stomach and esophagus (40, 47, 49, 50) (**Table 1**), however, the protease that activates GSDMA has not been elucidated, though by conducting protein-protein network (STRING-db) (86) analysis, it predicts caspase-1 (red stippled circle) has a strong relationship with GSDMA (**Figure 4A**). Some evidence suggested that GSDMA may participate in TGF-β production of gastric epithelial cell apoptosis, and gain-of-function mutations in this protein have been discovered to result in hair loss and keratosis (47, 87, 88). Human GSDMA has three mice orthologs, and the generation of knockout (KO) mice displayed increased susceptibility to invasive infection (89) (**Table 2**). GSDMA genetic polymorphisms indicates a connection with inflammatory bowel disease (IBD) and asthma in children (103–106). However, the gain-of-function mutations were discovered to impair the interaction between the NT and CT fragments by interfering with autoinhibition, causing constitutive activation and inducing pyroptosis without unleashing autoinhibition (88) (**Table 1**). Similar to GSDMD-NT, when the NT fragments of GSDMA and GSDMA3 were overexpressed in HEK 293T cells, they enhanced pore formation in the cell plasma membrane and trigger pyroptosis-like features (42). Other studies have shown that the NT of mouse GSDMA3 associates with the chaperone protein, heat shock protein 90 (HSP90) (107), and STRING analysis reveals a potentially functional link between GSDMA and heat shock-related 70 kDa protein 2 (HSPA2) (**Figure 4A**). Though there are no studies to date depicting a connection between GSDMA and protozoan parasites (**Table 1**), recent studies conducted on group A *Streptococcus pyogenes* (GAS) revealed that the GAS cysteine protease SpeB cleaves GSDMA to trigger pyroptosis in keratinocytes (52, 89). Upon SpeB cleavage, GSDMA unleashes its NT that forms pores in the plasma membrane which act as a conduit to release intracellular products simultaneously allowing extracellular SpeB to diffuse into the cell, resulting in more GSDMA cleavage and IL-1β processing. Notably, SpeB processes IL-1β at a different site compared to caspase-1, indicating a caspase-1 independent pathway to release IL-1β. In addition, mouse deficient in *Gsdma1* gene displayed blunt immune response to GAS, causing disseminated bacterial infection (52). It would be intriguing to determine if protozoan parasites could trigger the cleavage of GSDMA by activating proteases, and what are the consequences of forming GSDMA pores.

**Table 1:** Gasdermin family expression patterns and functions in protozoan parasite infection.

Human gene	Expression	Cleaved by	Biological function	Major diseases	Role in parasitic infection
*GSDMA*	Gastric, skin, tongue, esophagus, mammary glands, and umbilical cord (40, 50, 51)	Group A *Streptococcus* (GAS) cysteine protease SpeB (52)	Not identified	Alopecia, asthma, systemic sclerosis (47, 53, 54)	Not identified in parasitic infection. Deficiency in *Gsdma1* results in blunt immune response to GAS in mouse (52)
*GSDMB*	Airways, lymphocytes, esophagus, liver, stomach and colon	Granzyme A (55)	Not identified	IBD, asthma, type 1 diabetes (56–60)	Enhance the cleavage of GSDMD by caspase-4, implicating a role in parasitic infection (61)
*GSDMC*	Esophagus, intestines, bladder, keratinocytes, spleen, trachea (62–64)	TNFR-caspase-8, caspase-6 (65)	Not identified	Melanoma (66)	A substrate for caspase-8 and caspase-6, suggesting a potential to activate autophagy pathway and switch from apoptosis to pyroptosis (65)
*GSDMD*	Immune cells, esophagus, placenta, gastrointestinal tract epithelium	Inflammatory caspases, neutrophil elastase, cathepsin G, RIPK1-caspase-8 (19, 20, 67–69)	Pyroptosis and NETosis (69, 70)	Sepsis, experimental autoimmune encephalomyelitis, macular degeneration (71, 72)	Release alarmins and pro-inflammatory cytokines to eliminate extracellular *E. histolytica* (22, 73) and intracellular parasites (see **Table 3**)
*GSDME*	Placenta, brain, heart, kidney, cochlea, intestines, and IgE-primed mast cells	Granzyme B, caspase-3 (74, 75)	Pyroptosis (76)	Autosomal dominant nonsyndromic hearing loss (77–82)	May serve as functional analogous of GSDMD in lower vertebrate (83, 84), roles are not clearly identified in protozoan parasites
*DFNB59 (PJVK)*	Inner ear hair cells, auditory system, brain, eye, heart, lung, kidney, liver, testis (85)	Not identified	Hair cell maintenance, auditory pathway neurons activity	Recessive nonsyndromic hearing impairment (85)	Not identified

**Table 2:** Available experimental knockout mouse in gasdermin family members.

Gasdermin (GSDM)	Types of knockout mouse	Outcome and use of study
GSDMA	Triple knockout mice (three homologues in human) (89) Mouse that genetically deficient in *Gsdma1* (52)	Increased susceptibility to invasive infection by a pandemic hypervirulent M1T1 clone of human pathogen group A *Streptococcus* (GAS) (89). *Gsdma1* deficiency blunts mouse immune responses to GAS, causing extensive bacterial dissemination and detrimental to the host (52).
GSDMB	Restricted *in vivo* mouse model (57)	Due to the lack of a mouse homologues, unique in GSDM family (41).
GSDMC	Mouse *Gsdmc*1-4 cluster is confirmed by phylogenetic analysis (40) and established in mouse melanoma cells as an oncogene for melanoma progression (66, 90). Knocking down/out mouse *Gsdmc* 1-4 in B16 mouse melanoma cells (91).	Combined knocking down/out mouse *Gsdmc* 1-4 in B16 mouse melanoma cells results in reduced cell pyroptosis *via* triggering by dimethyl-α-ketoglutarate (DM-αKG) (91).
GSDMD	*Gsdmd^-/-^* mice are commercially available, were used and reported by two groups independently in 2015 (19, 30).	Based on various kinds of infection, *Gsdmd^-/-^* display more or less susceptibility to invasion comparing to wild-type mice (92–95). *Gsdmd^-/-^* mice are also used in behavioural test, and presented with increased anxiety (96). In experimental autoimmune encephalomyelitis (EAE) mouse model, *Gsdmd^-/-^* mice exhibit resistance from death and morbidity (71, 97).
GSDME	*Gsdme^-/-^* mice has been generated and deletion of GSDME in other cell types such as immortalized BMDMs, murine melanoma GSDME KO B16 cell line (98), and murine thymoma cell line EG70-Ova haven been reported (99).	GSDME deficiency promotes cell growth in cell culture and melanoma in mouse model (99). Melanoma cells that deficient in GSDME process larger tumors than wild-type cells (99).
DFNB59	*Dfnb59^-/-^* mice	*Dfnb59^-/-^* mice display hearing impairment (85, 100–102).

### GSDMB

At present, GSDMB remains the least studied. GSDMB is the most divergent member among the GSDM family dominantly located in airway epithelium, oesophagus, stomach, liver, small intestine and colon (108, 109). Human GSDMB have at least four isoforms with viable linkers that are cleaved by granzyme A from cytotoxic lymphocytes to induce target cell pyroptosis (55, 110). However, *Gsdmb* has not been found in rodents and this limits the development of experimental tools to study GSDMB in mouse models (**Table 2**). The granzyme A-GSDMB pyroptotic axis may also play important roles in immunity to cancers in addition to microbial infections (**Table 1**), however, no predicted interactions were found between them (**Figure 4B**). Genome-wide association studies (GWAS) uncovered a strong correlation between GSDMB and susceptibility to inflammatory diseases including Crohn’s disease, asthma, and type I diabetes (111–113). However, the direct relationship between GSDMB and these inflammatory diseases need to be further identified. A single nucleotide polymorphism (SNP) was discovered to associate with upregulated GSDMB expression and increased susceptibility in asthma (56). GSDMB is frequently expressed in cancer cells, consequently, significant correlations were indicated between expression levels of GSDMB and overall survival of patients with bladder carcinoma or skin cutaneous melanoma (55). More recently, a study showed that upregulated of GSDMB in IECs was associated with increased susceptibility of IBD (57) (**Table 1**). An essential physiological restoration and recovery mechanism to maintain intestinal homeostasis is the resolution of inflammation, and IECs play a major role to undergo proper tissue restitution and repair (114). Specifically, GSDMB was found to be significantly upregulated in IECs from active IBD patients as compared to healthy individuals, suggesting a strong correlation between GSDMB in IECs and IBD progressions (57) (**Table 1**). Unlike other GSDM members, GSDMB can bind to cell membrane even with the full-length protein (113). Another unique feature about GSDMB is its NT was found to directly interact with caspase-4 CARD protein which may have the capability to upregulate the cleavage of GSDMD mediated by caspase-4 (61). This study discovered that a reduction of GSDMB expression alleviated GSDMD cleavage and programmed pyroptotic cell death (61). GSDMB overexpression facilitated GSDMD cleavage along with elevated lactate dehydrogenase (LDH) release (61), indicating that GSDMB could act as a regulator for GSDMD cleavage in certain circumstances including protozoan parasite infection. In addition, since GSDMB can promote caspase-4 activity by directly binding to the CARD domain of caspase-4, this suggests that GSDMB can play a role in *Eh* pathogenesis as caspase-4 regulates GSDMD pore-forming activity in response to *Eh* (22).

### GSDMC

GSDMC, also known as melanoma-derived leucine zipper containing extranuclear factor, was named by its initial observation in melanoma metastases in mice (66). The expression of GSDMC is restricted to the esophagus, skin, spleen and vagina (109) (**Table 1**) and its expression was first used as a marker for melanoma progression (66). Upregulation of GSDMC was detected in human colorectal cancer tissues and was experimentally confirmed with its oncogenic potential (115). Human GSDMC is preferentially expressed in metastatic melanoma cells (66). Mice lack GSDMB but express GSDMC1, 2, 3, 4 (19) (**Table 2**). Knockdown of mGSDMC1-4 in B16 mouse melanoma was an accidental finding when dimethyl-α-ketoglutarate (DM-αKG) was used to treat HeLa cervical carcinoma cells (**Table 2**). The treatment of DM-αKG vastly triggered cell death, which presented with distinct morphology alterations in pyroptosis (91). Studies in Chinese Han population revealed that genetic polymorphisms of *Gsdmc* can alleviate the risk of Lumbar disc herniation, which is a common spinal disease (116). Like other GSDM family proteins, overexpressed GSDMC-NT can drive pore formation and cell pyroptosis in 293T cells (42). To date, no protease has been identified to initiate GSDMC cleavage (66) (**Table 1**). Based on STRING analysis (86), GSDMC interacts with LRRC57 (**Figure 4C**), and Synaptosome Associated Protein (SNAP23) was identified as the downstream of LRRC57 in human (117), indicating an interesting relationship between GSDMC and SNAP23. SNAP23 was found to be downregulated in *Eh*-induced hyperactivated macrophages (22), which may play a major role in repairing GSDMs pores on the plasma membrane. Delineating the interactions between GSDMC and SNAP23 could strengthen our understanding on the balance between cell hyperactivation and cell pyroptosis, and what triggers GSDMs to switch from a pyroptotic role to a non-pyroptotic dominant function. It was recently shown that GSDMC is specifically cleaved by caspase-8 following tumor necrosis factor α (TNF-α) stimulation under hypoxic conditions (65). TNF-α-induced GSDMC cleavage at Asp365, unleashing the GSDMC-NT to form pores and initiate cell pyroptosis in breast cancer cells (65). In *Eh*-stimulated macrophages, we detected bioactive IL-1β release in CRISPR-Cas9 gene edited *GSDMD KO* cells, suggesting that perhaps other GSDM pores (22) including GSDMC may have occurred to elicit robust pro-inflammatory responses.

### GSDMD

**Figure 5:**
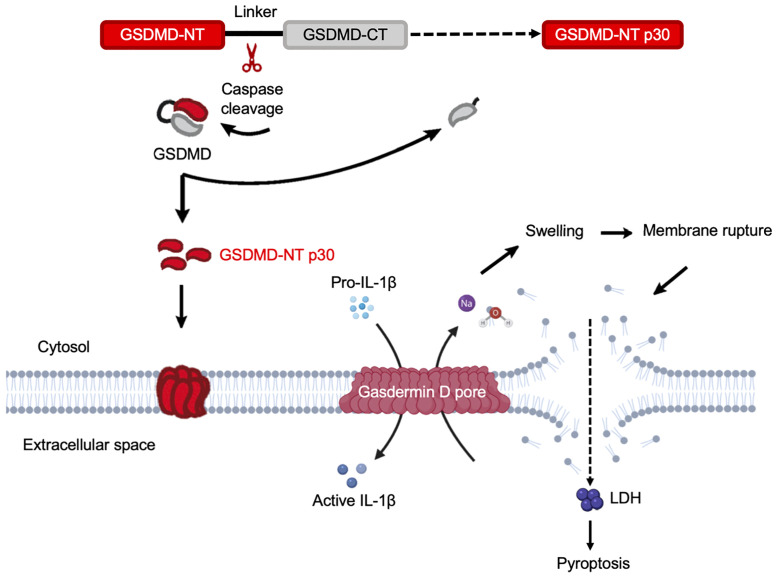
Gasdermin D functions as the executor of cell pyroptosis. Inflammatory caspases are activated by a wide array of inflammasomes upon sensing cytosolic contamination or perturbation. Upon activation, caspases cleave pro-IL-1β, pro-IL-18, and GSDMD. Full length GSDMD is composed of an NT effector domain, also known as pore-forming domain (red) and a CT repressor domain (grey). These two components are connected by a flexible link containing the caspase cleavage sites. The liberated NT pore-forming domain then inserts into the plasma membrane and triggers oligomerization of approximately 16 monomers to form a GSDMD pore. This is a relatively small pore allowing IL-1β to release through it. Simultaneously, sodium gets into the cell, bringing along water that increases cell volume. This can rapidly exceed the volume capacity of the cell, causing a big tear in the plasma membrane that is much larger in size than the GSDMD pore. This membrane-rupture event results in massive release of soluble cytosolic contents, including lactate dehydrogenase (LDH).

GSDMD is found in the gastrointestinal tract and in sentinel cells of the immune system, macrophages and dendritic cells (118). It was the first GSDM protein identified as the executor of pyroptosis that acts downstream of inflammatory caspases (19, 20). *Gsdmd^-/-^* mice has been widely used in research including inflammatory-caspase-associated autoinflammatory conditions and septic shock, and display protection from death as compared to wild-type counterparts (**Table 2**). By investigating polymorphisms in GSDMD, it was found that polymorphisms can dramatically impact GSDMD functions by affecting sites that are considered as structurally important (119). A prominent portion of GSDMD research has been focused on bacterial exposure, whereas *in vitro* studies in human and mouse macrophages discovered that *Eh* activates caspase-4 *via* noncanonical inflammasome pathway to regulate the cleavage of GSDMD to mediate IL-1β secretion (21), and STRING analysis (86) indicates strong interaction between GSDMD and caspase-4 (**Figure 4D**). GSDMD is the most well-characterized GSDM protein with its pore-forming NT effector that punches pores in plasma membrane. This discovery has shifted the paradigm of our understanding on how programmed cell death occurs (19). Remarkably, the effector molecule that induced pyroptotic cell death remained unknown until 2015 when GSDMD was discovered as the key effector of pyroptotic cell death and a cleavage target for caspase-1 and -11 (19, 20) (**Figure 4D**). The gene *Gsdmd* encodes the 480-residue protein GSDMD, which contains an NT and a CT domain (20, 120). Mechanistic studies demonstrated that GSDM-NT harbours the intrinsic pore-forming and pyroptosis-inducing activity (19, 20, 42). GSDMD are expressed as inactive forms in the resting state, where the CT fragment is held back in check with the NT component to trigger an intramolecular auto-inhibition to inhibit GSDMD cleavage (42, 121). Upon activation by cytosolic danger and infection sensors in macrophages and dendritic cells cleave GSDMD within the linker region, liberating the active NT pore-forming domain to assemble GSDMD pores in the cell membrane (19) (**Figure 5**). Recently, it was reported that GSDMD-CT not only auto-inhibits GSDMD-NT, but also provides a platform for recruiting inflammatory caspases and GSDMD activation (122). Thus, it is not surprising that mutations within the GSDM-CT region can impair the inhibitory interaction between the CT and NT domains that can trigger spontaneous cleavage of GSDMs. GSDM pores induce disruption in cell membrane integrity that ultimately leads to plasma membrane rupture and osmotic cell lysis, in which pro-inflammatory cytokines are released into the extracellular space (118, 123, 124) (**Figure 5**). Expression of GSDMD-NT itself can trigger inflammatory cell death, whereas overexpression of GSDMD-CT can inhibit pyroptosis (19). Immune cells release pro-inflammatory molecules into the extracellular space, therefore eliciting inflammatory and immune responses that play a role in innate host responses to parasitic infections. *Eh* as an extracellular protozoan parasite that induces outside-in signaling in human macrophages to activate caspase-4 similar to *Eh*-induced caspase-1 activation (22). Importantly, we recently shown that caspase-4 triggered GSDMD cleavage was more efficient in the release of IL-1β independent of the canonical NLRP3 inflammasome pathway while keeping the cells alive (22) (**Table 1**, see section below on intracellular protozoan parasites). By functional enrichment analysis on protein-protein interaction networks (86), GSDMD is intensively connected with both caspase-4/1 and NLRP3/1 inflammasomes. Humans with mutations in NLRC4 develops an autoinflammatory syndrome presented with acute fever and feature indicative of Macrophage Activation Syndrome (MAS). MAS patients present with elevated serum IL-18 as predicted by STRING analysis (**Figure 4D**).

A mounting body of evidence has demonstrated that neutrophils are crucial effector cells against *Eh* and some intracellular protozoan parasites (125–128) (**Table 1**). Inflammasome activation promotes neutrophil recruitment, which is critical to parasite dissemination and/or exacerbation of disease (129). In contrast to macrophages, GSDMD cleavage in neutrophils is caspase-independent, and is mediated by a neutrophil-specific serine protease, elastase (ELANE) released from cytoplasmic granules into the cytosol during neutrophil death (92). ELANE cleaves GSDMD to perforate the plasma membrane to trigger pyroptosis in neutrophils. GSDMD in neutrophils are linked to azurophilic granules and upon cleavage into GSDMD-NT, it transports to azurophilic granules that results in leakage of ELANE into the cytosol to cleave GSDMD, leading to a secondary cleavage on GSDMD (130). Neutrophil extracellular traps (NETs) catch and damage *Eh* and play an important role in innate host defense against infection (131). These findings highlight the principal differences between neutrophil and macrophage in GSDMD regulation that implicate neutrophil-specific functions in response to parasites (130).

### GSDME (DFNA5)

GSDME is variably expressed in a range of human cells and tissues, including the brain, endometrium, placenta and intestine (132) and was characterized as the causative gene for nonsyndromic hearing loss and as a tumor suppressor (40, 133) (**Table 1**). GSDME deletion by CRISPR/Cas9 gene editing in various cell types has been generated, for example, murine melanoma cell line B16 and thymoma cell lone EG7-Ova (99) (**Table 2**). *Gsdme^-/-^* mice exhibit healthier and more robust phenotypes after peritoneal injection of cisplatin comparing to wild-type mice (74) (**Table 2**). Other studies have reported gain-of-function in *Gsdme* leads to disruption of exon 8 at mRNA level and cause nonsyndromic DFNA5 hearing impairment (77–79, 134–137). It is not clear what the physiological role of GSDME is in these tissues and cells, where inflammation might be harmful (96). GSDME and pejvakin (PJVK) belong to the deafness-associated genes (DFN) and their protein sequences cluster together, distant from the other GSDMs (GSDMA-D). Evolutionarily, GSDME and PJVK are among the oldest GSDM members to date, with resemble sequences found in lower vertebrates and some invertebrates (138, 139). DFNA5 plays a role in the TP-53-regulated cellular response to DNA damage probably by cooperating with TP53 (140). Notably, GSDME was recently shown to be cleaved and activated by caspase-3 with a role in anti-tumor immunity (141, 142) (**Table 1**), driving chemotherapy drug-induced normal-tissue damage or viral infection-mediated secondary necrosis (74, 75) as confirmed by STRING analysis (86) (**Figure 4E**, **Table 1**). A recent study revealed that NLRP3 inflammasome activation causes delayed necrotic cell death by recruiting ASC in caspase-1/11 deficient macrophages (143). Additionally, ASC triggered caspase-8 activation to process GSDME, which is essential for caspase-1 independent necrosis without detectable IL-1β secretion, suggesting an alternative pathway for necrotic cell death regulated by NLRP3 inflammasome upon inhibition on caspase-1 (143). Furthermore, constitutive NLRP3 activation retains the capability to trigger inflammation in mice lacking GSDMD likely following GSDME-regulated pyroptosis (144). Thus, GSDME presents as a potential treatment for inflammatory disorders induced by sustained inflammasome activation (**Table 1**).

### DFNB59

DFNB59 or PJVK is a protein related to hearing impairment or loss in humans and mice (85, 145, 146). Since all GSDM family members share the two-domain structure, excluding DFNB59, it is a more remotely associated GSDM family member with a truncated non-homologous CT fragment (147) with no interaction with other GSDM family members (**Figure 4F**). DFNB59 mRNA is present in the brain, eyes, ears, heart, liver and intestines (85, 148) (**Table 1**). Even though GSDME and DFNB59 are associated with nonsyndromic hearing loss and impairment, *Dfnb59^-/-^* but not *Gsdme^-/-^* mice display hearing dysfunction (85, 100–102) (**Table 2**). Using *Pjvk KO* alleles, it was shown that PJVK was essential for regular mechanotransduction in hair cells (149). Missense mutations within the NT of DFNB59 results in impairment of transmitting the auditory signal through auditory neurons (**Table 1**), present either dysfunctional in cochlear outer hair cells (100) or auditory neuropathy (85). It is still not known if DFNB59 is a pore-forming protein or if it remains physiologically active under homeostasis, since the NT of DFNB59 is extremely short and might fail to trigger autoinhibition.

## Gasdermin D-Mediated Cell Pyroptosis

Pyroptosis (in Greek, *pyro* means fire and *ptosis* means falling) is a lytic form of regulated cell death that is induced by inflammatory caspase-1, 4, 5, and 11 (mouse) (150, 151), and was first reported as a cell biological phenomenon in macrophages during *Shigella flexneri* and *Salmonella enterica* serovar Typhimurium infections, respectively (152–154). GSDMD pores in the plasma membrane act as conduits through which low molecular weight cellular contents are released into the extracellular space to elicit inflammation. However, once the volume exceeds membrane capacity, a membrane rupture event occurs to release soluble cytosolic contents (19). This tear is robust enough to instantly dissipate soluble protein like LDH, while organelles are retained (155). During cell pyroptosis, the damaged membrane forms large ballooning bubbles and dying cells begin to flatten as their cytoplasmic contents are released (156). The rapidity of cell death and morphological alterations are signatures to distinguish pyroptosis and apoptosis (118). It is predicted that after the rupture event, the osmotic pressure achieves a new balance, and stops further volume increases (157). Once the pore-forming fragment is unleashed from the CT repressor domain, it subsequently oligomerizes to form the pyroptotic pores, in which an estimated 16 pore-forming fragment (NT) monomers oligomerize to form pores with a diameter of approximately 10-15 nm of range (42, 43, 45), or around 21 nm (44). These pores mediate the passage of cytokines and diverse cytoplasmic contents and undermine the cellular ionic gradients (**Figure 5**). The opening of GSDMD pore breaks the normal permeability barrier of the plasma membrane, resulting in membrane disruption, which causes an increase in osmotic pressure causing an influx of water, leading to the cell volume to increase (157). As a consequence, this drives cell swelling and plasma membrane rupture, which eventually lead to cell pyroptosis (**Figure 5**).

### NINJ1 Actively Mediates Plasma Membrane Rupture During Lytic Programmed Cell Death

It was originally thought that plasma rupture occurred after pore formation as a passive cellular event triggered by shear force (158). However, recent studies have shown that plasma membrane rupture is an actively regulated process that is mediated by a specific membrane protein, ninjurin-1 (NINJ1) (23). Deletion of *NINJ1* in bone marrow-derived macrophages (BMDMs), inhibited the release of HMGB1, a pro-inflammatory DAMP (159), despite exhibiting normal GSDMD-dependent release of IL-1β, indicating that the rupture of plasma membrane was an abnormal event. Macrophage’s deficiency in *NINJ1* displayed significant decreased in LDH release in the absence of GSDMD cleavage (23). IL-1β secretion was unaffected in *NINJ1^-/-^* BMDMs, supporting the notion that IL-1β is predominantly secreted through GSDMD pores.

NINJ1 is a 16 kDa cell-surface protein that contains two transmembrane regions with N and C termini exposing to the cytoplasm (**Figure 2**). With the use of artificial dyes of various sizes, it was revealed that NINJ1 acted downstream of GSDMD pores, and overexpressing NINJ1 in HEK 293T cells enhanced cytotoxicity (160). This finding was paradigm-shifting, as membrane rupture event has been long presumed to passively and spontaneously occur after cell death is triggered. Though much work needs to be done on the interaction between GSDMD and NINJ1, there are many unanswered questions: what drives the oligomerization to induce NINJ1 activation; what role does GSDMD play in regulating NINJ1 activity; what are the functions of the other NINJ family members and do they interact with each other and what is the biochemical activity of the NINJ family. More importantly, the link between NINJ1 activity in plasma membrane rupture and cytotoxicity requires more attention.

In terms of therapy, it is promising to target GSDMD or NINJ1 to limit inflammation rather than to simply block either NLRP3 or IL-1β, because the ultimate goal is to prevent pyroptosis in diseases such as autoinflammatory genetic disorders (97). In addition to this, a group of small molecules that target and inhibit GSDMD has been reported (161, 162). Because robust evidence indicates that NINJ1 as another new therapeutic target to limit inflammation. This could be achieved by either knocking out *NINJ1* or with an antagonistic anti-NINJ1 antibody, aimed at inhibiting NINJ1 to reduce inflammation and suppression of plasma membrane rupture (23).

### The GSDME/Caspase-3 Signaling Function as a Switch Between Apoptosis and Pyroptosis Against *E. histolytica* and Other Parasitic Infections

**Figure 6:**
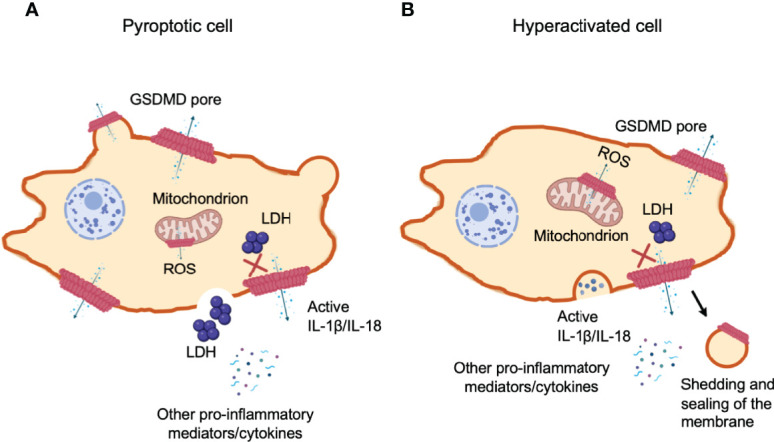
Gasdermin D-mediated cell hyperactivation. Cell pyroptosis and IL-1β secretion can be uncoupled under conditions of cell hyperactivation. This is a stage where phagocytes release IL-1β while remaining intact. The cleaved GSDM-NT fragments can also bind to mitochondrial membranes with higher affinity. NT pore-forming fragments of both GSDMD and GSDME permeabilize the outer membrane of the mitochondria, driving the generation of reactive oxygen species (ROS), loss of transmembrane potential and the release of cytochrome (*c*). **(A)** Pyroptotic cells form large balloon-like membrane structures and dying cells appear to flatten as their cytoplasmic contents are released, which is followed by cell swelling and membrane rupture. **(B)** Hyperactivated and pyroptotic cells can be differentiated by measuring lactate dehydrogenase (LDH) release, as it is too large to exit *via* GSDMD-NT pores and fully dependent on cell lysis for its release. Hyperactivated cells might have the capability to repair GSDMD pores through shedding of the disrupted membrane to recover damaged area. However, the mechanism to determine if GSDMD pores trigger cell pyroptosis or hyperactivation remains unclear.

Apoptosis constitutes a programmed non-lytic and non-inflammatory mode of cell death involving apoptotic executioner caspase-3, caspase-6 and caspase-7. Cell membrane integrity is preserved at early stages of apoptosis, and apoptotic cells are efficiently eradicated from healthy tissues by phagocytosis prior to being lysed. Classical apoptosis is a physiological programmed cell suicide in which cells undergo characteristic morphological changes, including membrane blebbing, cell shrinkage, DNA fragmentation, chromatin condensation (141, 142), whereas pyroptotic cells rapidly lose cell membrane integrity, increase in size, and have smaller nuclei by forming GSDM pores in which the damaged membrane forms large ballooning bubbles, which are of distinct morphological changes as apoptosis (156, 163–165) (**Figure 6A**). It is a well-defined step-wise process for *Eh* to kiss host cells to death *via* Gal-lectin contact with upregulation of intracellular Ca^2+^ levels followed by dephosphorylation of host proteins that activates caspase-3 to promote cell death (166). Blocking Ca^2+^ channels and/or Ca^2+^ chelators inhibit *Eh* killing of host cells (166, 167). *Eh*-induced apoptosis was found not to be dependent on caspase-8 or caspase-9 (168). *Casp-3 KO* mice are tolerant to amebiasis and a caspase-3 pharmacological inhibitor decreased *Eh* cytotoxicity (168, 169). Apoptosis is immunologically silent, thus by inducing apoptosis, *Eh* can dampen the inflammatory response to escape from being recognized.

Recent studies have significantly advanced our knowledge on the mechanisms and physiology roles of pyroptosis. Although pyroptosis and apoptosis have been considered as two distinct pathways, apoptotic stimuli can trigger pyroptosis under selective scenarios. GSDME is activated during classical apoptosis, following caspase-3 cleavage after Asp270 within the linker region (**Table 1**), which switches non-inflammatory apoptotic cell death into inflammatory pyroptotic death that express GSDME, alternatively, depletion of GSDME can switch apoptosis to pyroptosis in cells (74, 75). More specifically, activated caspase-3 can cleave GSDME to induce pyroptosis instead of apoptosis (74, 75). When GSDMs are activated by granzymes or caspases, it can also induce apoptosis in certain situations, where the expression of GSDMs is too low to induce pyroptosis, and vice versa, caspase and granzyme-induced apoptosis can be switched to pyroptosis by the expression of GSDMs (164). Another example is the discovery of the apoptotic initiator caspase-8 to interact with the adaptor protein ASC (170, 171). On the other hand, TNF receptor activation activates caspase-8 to induce GSDMD-dependent pyroptosis during pathogenic *Yersinia* infection (67, 68). Interestingly, activation of the inflammasomes drives the activation of apoptotic caspase-3/7 (172–175). Strikingly, deficiency of GSDMD remarkably postponed and decreased the amount of LPS-induced caspase-3 activation (75). The pore-forming fragment of GSDMD, but not full-length GSDMD, was discovered to cause cytochrome *c* release and caspase-3 activation (99), indicating that targeting at mitochondrial membrane, releasing cytochrome *c* and activating caspase-3 is a common mechanism employed by the gasdermin family members to potentiate apoptosis. At the onset of apoptosis, caspase-3 proteolytically cleaves GSDME, which is strongly implicated in tumor suppression (74). By inducing GSDME expression *in vitro*, it supresses colony formation and tumor cell proliferation in gastric cancer, melanoma and colorectal cancer and reduced metastasis in breast cancer (99, 176–178). Caspase-3 was shown to be involved in Jurkat T cell apoptosis induced by *Eh* through *in vitro* studies (168). *Eh*-induced apoptosis required contact *via* the Gal lectin but was independent of their cysteine proteases by applying the cell-permeable cysteine proteinase inhibitor, E64c. With the use of caspase-3 inhibitor Ac-DEVD-CHO, it was determined that caspase-3-like activity was necessary for *Eh* to trigger Jurkat cell apoptosis, since this inhibitor completely blocked Jurkat cell DNA fragmentation.

Transcriptomic analysis of mice brain that were infected with *T. gondii* compared to uninfected mice using RNA sequencing, showed that inflammasome-regulated pyroptosis was an important host cell death pathway to control infection (128, 179). By performing host-parasite interactome analysis, nine pyroptosis-related differentially expressed transcripts (DETs) were upregulated in infected mice, including *casp-4*, *gsdmd* and *pycard*, while *gsdme* was downregulated in chronic infection (180). Additionally, IL-1β and IL-18 were upregulated during chronic *T. gondii* infection, suggesting these inflammatory cytokines are crucial to assist mice to mount an appropriate inflammatory response to prevent latent *T. gondii* infection (180). During *T. gondii* infection, infected cells secrete growth factors to inhibit neutrophil apoptosis, by triggering Mcl-1 expression (181). Delayed neutrophil apoptosis may contribute to a robust pro-inflammatory response (181). *Leishmania* infection reduces caspase-3 activity and postpones spontaneous apoptosis in human neutrophils (182), serving as “trojan horses” to invade macrophages that are phagocytosing apoptotic cells (183). Clearly, more work needs to be done on how GSDM family members are involved as a master switch from apoptosis to pyroptosis, especially with protozoan parasite infections (**Table 1**).

## Gasdermin D Serves as a Gatekeeper to Release IL-1β in Hyperactivated Macrophages in Response to *E. histolytica*

When *Eh* contacts macrophage *via* the Gal-lectin adhesin, surface *Eh*CP-A5 RGD sequence binds α_5_β_1_ integrin that triggers Src family kinase phosphorylation and the opening of pannexin-1 channel. Pannexin-1 is a membrane channel for ATP that induces an extracellular burst of ATP, which engages the P2X_7_ receptor in an autocrine or paracrine fashion to activate the NLRP3 inflammasome and caspase-1 resulting in cell pyroptosis (184). By inducing the outside-in signaling in macrophage, *Eh* activates caspase-4 *via* the noncanonical inflammasome pathway to initiate IL-1β secretion similar to caspase-1 by keeping the cells intact (**Figure 2**) **(** 22). The mechanism of human caspase-4 activation requires live *Eh* in direct contact with macrophage to trigger outside-in signaling in macrophage to induce the generation of reactive oxygen species (ROS) and K^+^ efflux (21). Activation of pro-caspases undergoes dimerization or oligomerization with a succeeding cleavage into a small subunit and a large subunit. Upon being activated, it cleaves GSDMD to release the NT pore-forming domain that promotes pore formation to cause Na^+^ influx, cell swelling that ultimately triggers rupture in the plasma membrane. In response to *Eh* stimulation, caspase-4 also interacts with caspase-1 in a protein complex that potentiate the cleavage of caspase-1 CARD domains to enhance IL-1β secretion (21). More recently (22), we showed that *Eh*-induced IL-1β release was not due to massive cell pyroptosis, but rather induce macrophages to reach a stage of “hyperactivation” that caused sustained release of pro-inflammatory cytokines, revealing a significant non-pyroptotic role for GSDMD. This is in marked contrast to nigericin stimulated LPS-primed macrophages that activated caspase-1, resulting in massive cell pyroptosis (22). Mechanistically, *Eh* triggered caspase-4 activation, GSDMD cleavage and pore formation within 5 min and with IL-1β secretion as early as 30 min in the absence of cell death (22). An important finding was that caspase-4 cleavd GSDMD at the identical amino acid as caspase-1 to maximally regulate IL-1β secretion in response to *Eh*. *Eh*-induced IL-1β secretion was independent of pyroptosis as revealed by pharmacologically inhibiting GSDMD pore formation and in CRISPR-Cas9 gene edited *GSDMD KO* macrophages (22). We theorize that *Eh*-induced caspase-4/1 activation induced fewer GSDMD pores than NLRP3 inflammasome agonist such as LPS + Nigericin. If fewer GSDMD pores are generated, the cell might respond by initiating compensatory mechanisms to downregulate cell volume allowing for sustained IL-1β secretion while maintaining cell viability, and this may be a unique mechanism in the biology of *Eh* that could play a crucial role in disease pathogenesis and host defence.

It is controversial whether IL-1β is effectively released *via* pyroptotic membrane rupture or in the absence of pyroptosis (185–189). With intracellular pathogens, pyroptosis mediated by GSDMD was necessary for IL-1β release from macrophages exposed to inflammasome activators (155). Cell- and liposome-based assays demonstrated that GSDMD pores were required for IL-1β transport across an intact lipid bilayer (42–45). Of note, *GSDMD^-/-^* macrophages are still able to release caspase-1-processed bioactive IL-1β *via* PIP2-mediated plasma membrane binding that is of a much slower rate than GSDMD-dependent release (190). These findings identify a non-pyroptotic function for GSDMD, and raised the possibility that GSDMD pores represent conduits for the secretion of cytosolic cytokines under conditions of cell hyperactivation (191, 192).

### Gasdermin D-Regulated Cell Hyperactivation

How can a cell release IL-1β/IL-18 through GSDM pores in the absence of cell lysis? Secretion of the IL-1 family of cytokines was widely considered a caspase triggered pyroptosis-dependent event, as IL-1β and IL-18 lack a protein secretion signal sequence (193). Recently, the concept of “unconventional protein secretion” was advanced to describe GSDMs pore-mediated pro-inflammatory cytokine release (194, 195) and cellular alarmins such as ATP and high-mobility group box 1 (HMGB1) at the same time to augment inflammation in tissues to recruit immune cells against infection and damage. Phagocytes can achieve a state of hyperactivation, which is defined by their ability to secrete the IL-1 family of cytokines while retaining viability, but it remains unclear how IL-1β can be secreted from living cells (196).

When macrophages, dendritic cells and neutrophils survive inflammasome-mediated GSDMD pore-forming activity without membrane rupture and pyroptosis, they retain their ability to produce pro-inflammatory cytokines, termed “hyperactivated” cells (197). The hyperactivated cells and pyroptotic cells can be distinguished by measuring LDH in cell supernatants, which are too large to be secreted through GSDMD-NT pores and are only released upon cell lysis (**Figure 6A**). Mature IL-1 family cytokines and alarmin molecule HMGB1 are small proteins that are readily released through these pores. Alternatively, if the number of GSDMD pores patched in cell membrane exceeds the recovery capability of the cell, cell volume in turn increases. Once the volume exceeds membrane capacity, the plasma membrane divides from the cytoskeleton in large fluid-filled balloons. These pores disintegrate the cell membrane and are large enough to rapidly lose soluble proteins including LDH, whereas, organelles are retained (155). Shortly after the formation of GSDMD pores, a pyroptosis-associated membrane-rupture event proceeds which releases soluble cytosolic contents. Pyroptotic cells release both LDH and cytokines (196) (**Figure 6A**). Hyperactivated cells can process and pump out more inflammatory components as compared to their pyroptotic counterparts (141) (**Figure 6B**). At present, it still remains unclear what determines whether cleaved GSDMD causes pyroptosis or hyperactivation. Recently, researchers have proposed a possible mechanism of cell membrane repair response at the site of lesion, which efficiently recover membrane integrity, allowing cell survival. The battle between how promptly and efficiently the membrane repair response is initiated and how robust and fast the damaged is caused, is presumably dependent on how much and how efficiently GSDMD is cleaved.

### Mechanisms of Pyroptotic Membrane Repair Regulation

**Figure 7:**
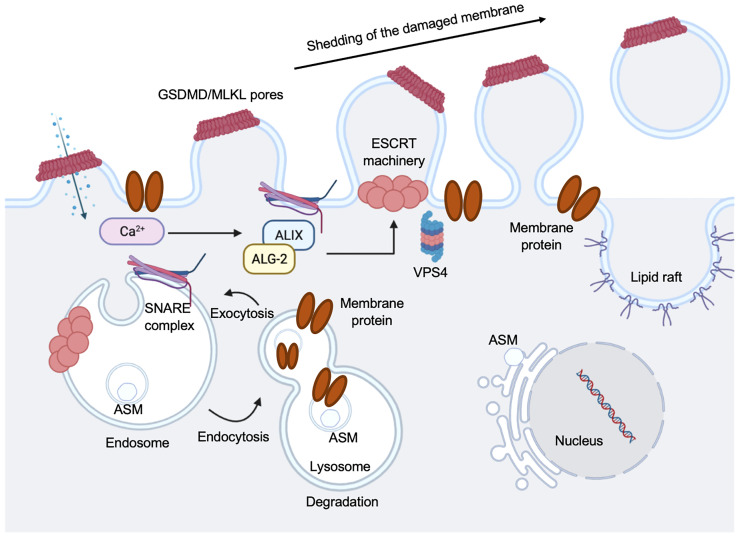
Regulation of pyroptosis membrane repair mechanisms. Pyroptotic pores formed by GSDMD, or necroptotic pores formed by mixed lineage kinase domain-like protein (MLKL), triggers ion exchanges across the plasma membrane. Ca^2+^ influx occurs through plasma membrane wounds, which in turn binds to apoptosis linked gene 2 (ALG-2) facilitating its recruitment and migration to the damage site. Elevation in the intracellular Ca^2+^ concentration triggers exocytosis of lysosomes. Lysosomal acid sphingomyelinase (ASM) is transported to the outer leaflet of the membrane. This is followed by the recruitment of ALG-2 interacting protein X (ALIX) and this occurs in an ALG-2 and Ca^2+^ dependent manner. This initiates ESCRT-III assembly, leading to membrane deformation into the extracellular milieu. A promising candidate is SNARE-mediated exocytosis to facilitate vesicle and plasma membrane fusion events. Vacuolar protein sorting-associated protein 4 (VPS4) leads to membrane repair and shedding of the damaged part of the cell membrane, recycling ESCRT subunits during this process.

Cells that have GSDM pores on plasma membrane can be sensed by an instantaneous increase in intracellular Ca^2+^ to trigger membrane repair by recruiting the endosomal sorting complexes required for transport (ESCRT) machinery to disrupted membrane areas and repair them in ectosomes (198) (**Figure 7**). Mammalian cells can repair plasma membrane damages when size of the disruption is not large (<100 nm) by a variety of mechanisms (199). Cell membrane that are damaged by GSDM pores likely induce activation of the ubiquitous plasma membrane damage repair response in eukaryotic cells, restoring membrane integrity rapidly to increase cell survival (200). The two major well-defined mechanisms are: Acidic Sphingomyelinase (ASM)-dependent endocytosis of plasma membrane pores and ESCRT-mediated shedding of injured plasma membrane (**Figure 7**). The ESCRT machinery was initially found in yeast, where it was identified to regulate protein trafficking and intraluminal vesicle formation at the distal side of cell membrane (201). The ESCRT machinery is a multiprotein complex contained a group of subcomplexes, including ESCRT 0, I, II, III, and ESCRT-III was discovered to be essential in membrane repair process (202, 203). The pyroptosis membrane repair involves massive endocytosis of damaged membrane, mostly from lysosomes (204) and exocytosis by promoting lipid raft formation (205, 206) (**Figure 7**). We have shown that when *Eh*CP-A5 engages α_v_β_3_ integrin on goblet cells, it facilitated outside-in signaling cascade by activating SRC family kinase, resulting in the activation of several kinases including PI3K and PKC (18). The trafficking vesicle marker, myristolated alanine-rich C-kinase substrate (MARCKS), was the target for PKC and has been implicated in docking vesicles for SNARE-mediated membrane fusion. We discovered that the R-SNARE vesicle-associated membrane proteins 8 (VAMP8) expressed on mucin vesicles was critical for SNARE-mediated exocytosis of mucin secretion in response to *Eh* (18). Thus, SNARE complex in goblet cells regulates mucin exocytosis and degradation of these regulatory pathways aggravates *Eh* pathogenesis (18). After the membrane is damaged and the uncontrolled entry of Ca^2+^ occurs, lysosomes are recruited to the site of the lesion and subsequently fuse to the plasma membrane, releasing ASM that hydrolyzes sphingomyelin into ceramide. This in turn, facilitates endosomes formation that internalize the lesion and repair membrane integrity (206–209) (**Figure 7**). A group evaluated the involvement of ASM in the repairing plasma membrane that caused by *Eh*. They found that damage to *Eh* membrane promotes lysosomes migration to the site of impairment where lysosomal ASM is released, forming membrane patches and endosomes to incorporate the lesion area and repair cell membrane, thus this mechanism facilitates amoebic viability (210).

Ca^2+^ influx through GSDMD pores acts as a signal for cells to undergo membrane repair by recruiting the ESCRT machinery to the site of damage or ruptured membrane (198) (**Figure 7**). Inhibiting the ESCRT-III assembly robustly favors pyroptosis in mouse BMDMs (198), suggesting a crucial anti-inflammatory role for the ESCRT-III machinery to restore cell homeostasis after GSDMD pore formation (**Figure 7**). Imaging the disruption of the electrochemical gradient using the Ca^2+^ dye Fluo-8, and loss of membrane integrity using propidium iodide (PI) in mouse BMDMs, showed that the ESCRT system dampens GSDMD pore-forming activity and downregulated cell death and IL-1β secretion by acting downstream of caspase activation and GSDMD oligomerization (198). However, it remains vague whether ESCRTs could serve as a control of caspase activation to regulate GSDMD activity, and if other GSDMs are involved in the process. ESCRT-III was discovered to repair mixed lineage kinase domain-like protein (MLKL)-induced plasma membrane injury in the process of necroptosis, indicating this membrane repair process can increase cell viability (triggering hyperactivation instead of pyroptosis) to combat the pore-forming activity of GSDMD (198) (**Figure 7**). The mechanisms on if bioactive IL-1β is packaged into micro-vesicles to be released or they are secreted primarily from the GSDMD pores remain to be elucidated. It is still unclear if these membrane repair processes occur in cells with GSDMD-disrupted plasma membranes.

## Gasdermin Pores Target Mitochondrial Membrane to Eradicate *E. histolytica* and Other Protozoan Parasites

**Figure 8:**
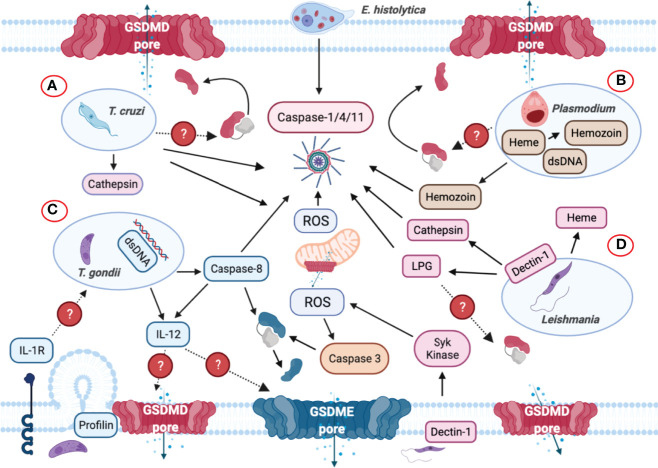
Inflammasome and GSDMs regulation by intracellular protozoan parasites. **(A)** *Trypanosoma cruzi* triggers NLRP3 inflammasome activation when the parasite lyses the parasitophorous vacuole to gain access to the cell cytoplasm. Cathepsin and reactive oxygen species (ROS) are involved in NLRP3 inflammasome activation. **(B)** *Plasmodium*-derived molecules such as hemozoin and dsDNA are released to the host cell cytosol upon lysosomal rupture and cathepsin release that trigger activation of the NLRP3 inflammasome. dsDNA can trigger NLRP3 inflammasome activation *via* the generation of ROS and membrane disruption. It is unclear if GSDMD or other GSDMs are involved in inflammasome-mediated IL-1β/IL-18 secretion. **(C)** Though *Toxoplasma gondii* is widely considered as a silent parasite, NLRP3 inflammasomes are activated upon infection. The parasite can be recognized by TLR-dependent sensing of *T. gondii*-derived molecules in murine dendritic cells that induces the production of IL-12. The identification of the parasite profilin as a PAMP mediates the recognition of *T. gondii*. Subsequently, the production of ROS is also elevated upon sensing this parasite but it remains unknown how GSDMD cleavage occurs during infection. Caspase-8 was discovered to mediate *T. gondii* control through innate production of IL-12, and IL-12 may be released *via* GSDMD/GSDME pores. Caspase-8 can induce the cleavage of both GSDMD and GSDME and act as the upstream regulator for caspase-3. **(D)** Upon phagocytosis by macrophages, *Leishmania* triggers Dectin-1, a C-type lectin receptor that signals containing the participation of Syk kinase, which in turn produces ROS to promote NLRP3 inflammasome activation. Additionally, *Leishmania* lipophosphoglycan (LPG) triggers caspase-11 activation, promoting NLRP3 inflammasome assembly *via* the noncanonical pathway. During this process, mitochondria generate ROS, lose their transmembrane potential and release cytochrome *c* into the cytosol, which subsequently activate caspase-3 to cleave GSDME or GSDMD, causing pyroptosis instead of apoptosis.

GSDM-NT can bind and insert into internal cellular membranes (70, 99, 107, 130), including mitochondria and bacteria to kill the cells (43). However, bacteria are known to survive during pyroptosis (155, 211) so the physiological relevance of bacterial membrane-targeting remains unclear. The NT of GSDMD and GSDME when translocated to mitochondria, permeabilize the outer membrane and destroy their function, resulting in the production of ROS (**Figure 2**, **Figure 6** and **Figure 8**), loss of mitochondrial membrane potential and the release of cytochrome *c*, which subsequently activates caspase-3 to augment cell death (99). For example, bacteria that remain viable during pyroptosis but are damaged may drive by the formation of GSDMD pores (43) and ROS (212). ROS and the release of mitochondrial DNA, mitochondria, nuclei, and ASC specks *via* cell membrane rupture and Ca^2+^ influx and K^+^ efflux through GSDM pores can trigger activation of inflammasomes (213). Noncanonical signaling pathway primarily triggers GSDMD cleavage and induces pyroptosis, however, activation of noncanonical signaling pathway can trigger assembly of the canonical NLRP3 inflammasome, possibly *via* the generation of mitochondrial ROS and K^+^ efflux, implicating a certain degree of crosstalk between these two pathways (214–216) (**Figure 2**). The noncanonical caspase-4 activation in response to *Eh* involves K^+^ efflux and ROS generation that can play an important role not only in amplifying downstream pro-inflammatory responses (**Figure 2**), but by interacting with the canonical NLRP3 inflammasome pathway to enhance the cleavage of caspase-1 CARD proteins (21). K^+^ channels are the most common identified ion transporter, and their critical roles was determined from colon biopsies from human with amebiasis with the demonstration of restrained K^+^ channel expression. Blocking K^+^ channels with genetic silencing or pharmacologic inhibitors suppressed caspase-1 activation, IL-1β secretion and cell pyroptosis in macrophages (217). The production of ROS is dispensable in killing *Leishmania amazonensis* but plays a major role in regulating inflammatory responses by regulating neutrophil infiltration into lesions (218). Activated phagocytes elicit cytotoxic impacts through ROS generation to kill pathogens by oxidative damage in Chagas disease causing *Trypanosoma cruzi* (219–223).

## Pyroptosis Defends Against Intracellular Protozoan Parasites

Intracellular protozoan parasites reside in neutrophils, dendritic cells and macrophages. GSDMD-mediated pyroptosis can effectively damage intracellular parasites-residing niche, releasing cytosolic contents to promote pathogen expulsion. This in turn, triggers a strong inflammatory response to eliminate the compromised cell. Removing the intracellular replicative niche is beneficial to the host, however, it raises the interesting question, how does pyroptotic cells or infected macrophages ultimately eradicate pathogens?

### Trypanosoma cruzi

*Trypanosoma cruzi* is an intracellular protozoan parasite that causes Chagas disease, which manifests as fever, fatigue, and headaches during early stage of infection, with potential to progress to heart failure and severe gastrointestinal complications (224–226). During the acute phase of infection, *T. cruzi* in circulating blood results in fever and swelling around the site of infection (227). Innate and adaptive host immune responses are both indispensable to control *T. cruzi* infection (228, 229). Upon lysing the vacuolar membrane, *T. cruzi* gains access to the host cell cytoplasm, where it undergoes replication (**Figure 8A**). Innate immune sensing by the NLRP3 inflammasome critically contribute to eliminating *T. cruzi* from healthy tissue (230, 231) (**Table 3**). *NLRP3^-/-^, caspase-1^-/-^* and *Asc^-/-^* mice display more severe parasitemia (231) and lower survival probability (230) compared to wild-type mice. Cathepsin B is essential for NLRP3 activation in response to *T. cruzi*, and pharmacologically inhibiting cathepsin B abolishes IL-1β release (231) (**Figure 8A**). Interestingly, NLRP3 deficient macrophages are not only presented with impaired IL-1β secretion but also nitric oxide (NO) release that causes increase in parasite replication (231). NO produced by innate immune cells is trypanocidal to control *T. cruzi* replication (246). Macrophages serve as first responders by activation of NAD(P)H oxidase (NOX2) and ROS production to eliminate *T. cruzi* (247) (**Figure 8A**). NOX2 and ROS promote T cell-mediated adaptive immune response and deficiency in them causes defective splenic activation of cytotoxic T cell immunity against *T. cruzi* invasion (248). By infecting p47^phox-/-^ macrophages with *T. cruzi in vitro*, NOX2 and ROS were discovered to regulate cytokinopathy through controlling cytokine gene expression. Cytokinopathy was demonstrated as a molecular mechanism in cardiomyopathy in Chagas’ disease by cardiac gene expression profiling (249). Under situations like trauma, infection, and inflammation, macrophages become activated and secret robust level of inflammatory cytokines including IL-1 and IL-6, triggering the cascade that results in cytokinopathy (250). However, with deficiency of NOX2, CD8^+^T cell generation and activation were compromised, causing parasite burden, tissue damage and death (248).

**Table 3:** The signaling and functions of gasdermin proteins defend against intracellular protozoan parasite infection.

Intracellular protozoan parasites	Activating stimuli and inflammasomes	GSDMs involved	Functions of GSDMs
*Trypanosoma cruzi*	NLRP3 inflammasome, K+ efflux, ROS, and cathepsin (230, 231)	Not fully identified. GSDMD and GSDME could be involved	IL-1β and IL-18 are crucial for parasite elimination through the generation of NO. GSDMD/GSDME-mediated pyroptosis might be required in eradicating the parasite, autophagy pathway could be the center of investigation (232, 233)
*Plasmodium* spp.	NLRP3 inflammasome, K+ efflux, ROS, cathepsin, Hemozoin and dsDNA (AIM2 inflammasome) (234, 235)	Not fully identified. GSDMD could be involved	Not clear if GSDMD or other GSDMs are cleaved and if cytokines are secreted *via* GSDMD pores or it is a pyroptosis-dependent event or if other GSDMs might be required. Experimental cerebral malaria mouse model indicates caspase-8 is highly activated and GSDMD may also involve (236)
*Toxoplasma gondii*	NLRP3 inflammasomes, K+ efflux, ROS, and ATP (170, 237, 238)	Not fully identified. GSDMD and GSDME could be involved	Caspase-8 and caspase-3 were shown to regulate GSDME cleavage. More work is required to determine how *T. gondii* regulates caspase-8 activity and if IL-12 is released through GSDMD/GSDME pores (68, 239–241)
*Leishmania* spp.	NLRP3 inflammasomes, K+ efflux, ROS, cathepsin and lipophosphoglycan (LPG) (noncanonical caspase-11) (242–244)	Not fully identified. GSDMD could be involved	LPG triggers the activation of caspase-11 to promote GSDMD pore formation. GSDMD cleaved by RIPK1 and RIPK1 kinase activity is required for IL-1β expression, but further investigation is needed to detect if cleaved GSDMD can mediate IL-1β and IL-18 secretion (244, 245)

GSDMD pore formation from NLRP3 inflammasome activation induces autophagy and may serve as a valuable anti-parasitic mechanism to eliminate *T. cruzi* from the host (251) (**Table 1** and **Figure 8A**). By studying the interplay between inflammasome and autophagy in *T. cruzi*-infected mice and peritoneal macrophages, it was demonstrated that autophagy governs trypomastigotes outcome and activity at early stages of invasion by forming the NLRP3-dependent autolysosomes (252). NLRP3 inflammasome is also a requirement for triggering functional autophagy in *T. cruzi* activated macrophages (252) (**Table 3**). Autophagy and autophagy associated proteins not only regulate excessive inflammatory responses (232) but also target intracellular pathogens and organelles (253). Thus, deficits in autophagy molecules can result in uncontrolled inflammasome activation and subsequent immunopathology (254, 255). Autophagy disrupts pyroptosis by downregulating GSDMD cleavage (256) (**Table 3**). A recent study revealed that inhibition of mammalian target of rapamycin (mTOR) signaling upregulated NLRP3 expression, ROS generation and IL-1β release that boosted the efficiency of parasite control in macrophages (257) (**Table 3**); however, the direct effect and connection between autophagy and rapamycin treatment are unknown (257). Another study reported that rapamycin as an autophagy agonist, prevents GSDMD-mediated pyroptosis after LPS treatment (258). Autophagy was demonstrated to coordinate GSDME-induced pyroptosis by utilizing chloroquine to block autophagy pathway in human melanoma cells following an unclear mechanism (233). It will be of interest to uncover the interplay between autophagy and GSDMD/GSDME to shed light on the role of GSDMs in *T. cruzi* infection (**Table 3**).

### Plasmodium

Malaria is a parasitic disease caused by the protozoa pathogens of the *Plasmodium* genus, which accounts for 228 million infected people globally in 2018 (259). Transmission occurs primarily through the bite of an infected female *Anopheles* mosquitoes, which inoculate the highly infective sporozoite forms into the mammalian host (260, 261). Different species can result in various clinical outcomes. This parasite has a unique life cycle in which sporozoites enter circulation and liver, where they undergo replication and differentiation (262). Upon exiting infected hepatocytes, red blood cells (RBCs) are infected where the parasites transform into trophozoites (263). Excessive activation of inflammatory responses in malaria causes a pro-inflammatory cytokine storm that coincides with severe fever episodes in patients that can be detrimental to the host. The cytokine storm contains IL-1β, TNF-α and IL-12 (262, 264), which effectively can control parasite replication (262). The release of inflammatory effectors is the consequence of hemozoin (265) (**Figure 8B**). The majority of the liver damage in malaria is an outcome of high oxidative stress accumulated by heme and infiltrated neutrophils regulated by NF-κB (266). When *Plasmodium* digests hemoglobin it converts free heme into an insoluble crystalline manner named hemozoin. Hemozoin drives inflammation in both sterile and infectious conditions, contributing to the pathogenesis of hemolytic disorders, including sickle cell disease and malaria (267, 268). Since the generation of hemozoin is a critical step for *Plasmodium* survival, it becomes a promising target for antimalarial drug development (269).

In the parasite vacuole, some *Plasmodium*-derived specific molecules, including hemozoin-derived from RBCs, parasite double-stranded DNA (dsDNA) and cathepsin are released in the cytoplasm to trigger NLRP3 inflammasome activation (234, 235) (**Figure 8B**). Sufficient NLRP3 inflammasome activation requires K^+^ efflux and NADPH oxidase induced by hemozoin in *P. berghei* ANKA sporozoites, suggesting NLRP3 as an essential player in cerebral malaria in mice, while not controlling parasitemia (270). By dissecting the mechanism of hemolysis, it was found that hemozoin triggered NLRP3 inflammasome activation in LPS-primed macrophages to promote IL-1β release (271). In addition, hemozoin-induced sterile hemolysis was demonstrated to trigger ROS production, tyrosine kinase (Syk) and NADPH oxidase activation that was crucial to inflammasome assembly (271). It is not known if this mechanism is involved in malaria pathogenesis. Conversely, NLRP3 was found to have a toxic impact in malaria, as mice deficiency in inflammasome components had higher survival rate with a lethal dose of *P. chabaudi* (234).

Caspase-8 was reported to be a primary mediator of systemic inflammatory response in *P. chabaudi* infected mice and in *P. berghei* experimental cerebral malaria. Knockout of caspase-8/1/11 or caspase-8/GSDMD resulted in disruption of TNF-α and IL-1β production, uncovering a supplementary and indispensable role for caspase-8 and GSDMD in malaria pathogenesis (236). The role of caspase-8 on IL-1β release needs more investigation as it is was shown to be involved in the cleavage of GSDMD (67). However, none of these roles in malaria pathogenesis has been addressed to date. It is not known whether these cytokines are secreted through GSDMD pores or it is a pyroptosis-dependent event or if other GSDMs might be involved (**Figure 8B**). A more comprehensive understanding using different *Plasmodium* species and in different models of disease would be beneficial to dissect the mechanisms on progression and regulation of malaria.

### Toxoplasma gondii

*Toxoplasma gondii* is an obligate intracellular parasite and is the causative agent of toxoplasmosis (272). In most, *T. gondii* infection remains asymptomatic, whereas devastating diseases can occur in immunosuppressed individuals, causing infections in the brain and other tissues (273). As an apicomplexan, *T. gondii* has evolved specialized secretory organelles, including the rhoptries (ROPs) and dense granules (GRAs), which are designated to inject effector molecules into the host cell (274). The parasite can also invade and replicate in host cell by secreting ROP effector proteins and building a parasitophorous vacuole (PV) (**Figure 8C**), with the injection of GRA molecules into the cell cytosol to promote parasite growth (275). Profilin was identified from *T. gondii* as a PAMP recognized by murine Toll-like receptor (TLR)11/12 (276–278) (**Figure 8C**). Innate detection of *T. gondii* induces IL-12 production in both dendritic cell and macrophage to directly sense and recognize parasite-derived molecules (**Figure 8C**). *Ripk3^-/-^Casp8^-/-^* mice succumbed to invasion with *T. gondii* that was rescued with exogenous IL-12 (239). Caspase-8 is an essential regulator of cell apoptosis and is indispensable for optimal transcription of inflammatory defense genes such as *il12* and *il1β* (239). Caspase-8 is an upstream regulator of caspase-3 that control cell apoptosis, while preventing RIPK3-MLKL-dependent necroptosis (**Figure 8C**). By using a small-molecule inhibitor to block TGFβ-activated kinase 1 (TAK1), the activation of caspase-8 was discovered to induce both GSDMD and GSDME cleavage in murine macrophages, causing cell pyroptosis (68). Additionally, caspase-8 was found to initiate NLRP3 inflammasomes in macrophages (240) that might serve as a compensatory pathway when caspase-1 activity is inhibited (170, 237). More work needs to be conducted on how *T. gondii* regulates caspase-8 activity and if GSDMD/GSDME pores mediates IL-12 release during this process (**Figure 8C** and **Table 1**).

*T. gondii*-induced macrophage death occurs *via* a guanylate binding protein (GBP) dependent event independent of GSDMD to promote apoptosis (279). GBP1 was found to be targeted in *Salmonella*-containing vacuoles to facilitate caspase-4 recruitment causing its activation and pyroptosis. These finding suggested an immune role for GBPs as a conduit for not only pyroptosis, but also apoptosis (279). *T. gondii* stimulation in human monocytes stimulates the Syk-CARD9/MALT-1-NF-κB signaling pathway and activation of the NLRP3 inflammasome to release IL-1β in a cell death- and GSDMD-independent manner (280). In contrast, *T. gondii* infection in microglia elicits NF-κB signaling to mediate pro-inflammatory cytokine secretion in innate host defence. Other studies have shown that GSDMD dependent IL-1α but not IL-1β release in microglia impaired parasite control and dysregulated immune cell infiltration. Unlike IL-1β, IL-1α functions as an alarmin molecule directly released without processing upon sensing invasive signals, acting as a rapid initiator in inflammatory response (281). IL-1α and IL-1β both process through the same receptor (IL-1R) (282) (**Figure 8C**). It was concluded that microglia act differently from macrophages as they can release the alarmin IL-1α to promote neuroinflammation and parasite control in *T. gondii* infection though they are present in the identical microenvironment in the central nervous system (241). Furthermore, IL-1R1 is primarily expressed on blood vasculature in the brain, and the pro-inflammatory response triggers in the brain during chronic *T. gondii* infection is regulated through IL-1α, but not IL-1β, since brain-resident microglia lack an NF-κB signature compared to monocyte-differentiated macrophages (241). In *GSDMD KO* there is a robust increase in *T. gondii* cyst burden six weeks post infection compared to wild-type mice, indicating an inflammasome-mediated neuroinflammation requires GSDMD to control *T. gondii* in the brain (241). However, studies are still needed to determine what role IL-1R could play in controlling *T. gondii* infection in the gut and macrophages. Activating the P2X_7_ receptor with extracellular ATP in *T. gondii*-stimulated macrophages enhanced parasite clearance by producing ROS and lysosome fusion with parasitophorous vacuole, forming a phagolysosome (238). Extracellular ATP also activated the NLRP3 inflammasome to potentiate IL-1β secretion by recruiting caspase-1. IL-1β released in the extracellular milieu binds to its receptor accelerating *T. gondii* eradication through mitochondrial ROS production (**Figure 8C**). *T. gondii*-mediated activation of the inflammasome results in caspase-1-mediated processing of IL-1α, IL-1β and IL-18 and pyroptosis (**Figure 8C**).

### Leishmania

Leishmaniasis is a vector-borne parasitic inflammatory disease caused by the protozoan parasite of the *Leishmania* genus. This disease affects millions of people, over 88 countries worldwide, especially prevalent in tropical and subtropical areas of the world (283). Self-healing cutaneous and debilitating visceral leishmaniasis affect approximately 1.5 million people globally (283, 284).

The parasite exists extracellularly as flagellated promastigotes in sand flies (283). *Leishmania* spp. interact with a range of cells, including neutrophils, monocytes, and macrophages, where the parasites differentiate into amastigotes and replicate inside the parasitophorous vacuole (283) (**Figure 8D**). Inflammasome activation is a signature event of leishmaniasis and is protective and responsible for the restriction of parasite replication in macrophages (242, 285). All different species of *Leishmania* can activate the NLRP3 inflammasome *via* K^+^ efflux and cathepsins to release IL-1β (242) (**Figure 8D**). Upon phagocytosis, the parasite turns on the C-type lectin receptor: Dectin-1, which elicits a Syk-dependent signaling pathway that results in ROS generation to contribute to NLRP3 inflammasome activation (243) (**Figure 8D**). More recently, it was shown that *Leishmania* lipophosphoglycan (LPG) can trigger the activation of caspase-11 to promote GSDMD pore formation, IL-1β secretion and pyroptotic cell death (244) (**Figure 8D**).

Receptor interacting protein kinase 1 (RIPK1) is a critical kinase that mediates necroptotic cell death following activation of various cell death receptors and TLRs (245). Human visceral leishmaniasis displayed increased serum levels of heme (**Figure 8D**), which is generated by hemoglobin catabolism, and is a potent stimulator of necroptotic macrophage (245). By examining the correlation between heme and necroptosis, it was found that heme strongly prevented *Leishmania* replication in BMDMs, and blocking RIPK1 kinase activity upregulated *Leishmania* replication without the presence of heme (245). Since GSDMD can be cleaved by RIPK1 (**Table 1**) and RIPK1 kinase activity is required for IL-1β expression in response to *Leishmania* (245), it would be of interest to detect if any crosstalk between necroptosis and pyroptosis exists in the presence of *Leishmania*. In addition, how pyroptosis affects parasite survival and virulence remains to be elucidated. Interestingly, it was reported that the host cell secretory pathway can export *Leishmania* zinc-metalloprotease GP63 and LPG (286) out of the vacuole, suggesting these virulent factors could act as the upstream regulator for caspase-11 activation. On the contrary, *Leishmania* can negatively regulate inflammasome activation by using GP63 to block PKC signaling in human monocytes, avoiding ROS generation, inflammasome activation and IL-1β production (287).

## Concluding Remarks

A protective role of inflammasome signaling and inflammatory cytokine release has been determined against protozoan parasites, particularly in the acute phase of infection. It is worth noting that in the past several decades new modes of regulated cell death have emerged to involve inflammatory form of necroptosis and pyroptosis, and other inflammatory manners of caspase-independent programmed cell death. Inhibiting pyroptosis ameliorates disease, including septic shock and autoinflammation, conversely, it can be detrimental for infections. The key roles of GSDMs in autoimmune and inflammatory diseases, infection, deafness and cancer are evolving, which reveals possible novel therapeutic avenues (76, 113, 288, 289). However, of note, the non-pyroptotic function of GSDMD has been uncovered in *Eh*-induced hyperactivated macrophages (22), and in GSDMD deficient LS174T cells (goblet cell line) that displayed vigorous reduction in MUC2 secretion when stimulated with mucus secretagogues such as ATP, histamine and PMA (290), suggesting a significant non-pyroptotic role of GSDMD in regulating cortical actin cytoskeleton disassembly during mucin granule exocytosis (291). By proteomics analysis, we determined SNAP23 is highly involved in membrane trafficking and is downregulated in *Eh*-induced hyperactivated macrophages (22), implying potential relevance may exist between exocytosis (membrane repair response) and conversion between hyperactivation and pyroptosis in macrophage. Mechanistically, if GSDM pores are effectively repaired by membrane repair pathways, pyroptotic cells might transform into non-pyroptotic or hyperactivated counterparts, whereas if GSDM pores keep releasing inflammatory cytokines and ions, the cell would finally burst. Thus, studying the mechanisms of the membrane repair system might shed light on defining the non-pyroptotic roles of GSDMs in the pathogenesis of protozoan parasites.

Pyroptosis and the connection between pyroptosis and apoptosis need further investigation. Caspase-3 as the common main protease that triggers the cleavage of GSDME (65) is the key determinant of which mode of programmed cell death pathway the cell follows. GSDME can be exploited as a master switch molecule in shifting between apoptosis and pyroptosis. The level of GSDME expression determines the fate of the cell: cells that express adequate GSDME trigger pyroptosis, while cells expressing insufficient GSDME elicit apoptosis (74). Moreover, GSDME cleavage particularly depends on caspase-3 and not caspase-7 to switch TNF-induced apoptosis to pyroptosis in HeLa cells (74). Since GSDME enhances caspase-3/7 activation in apoptotic cell death through targeting the mitochondria to release cytochrome *c* (99), we predict an inflammatory role of GSDME to trigger pyroptotic cell death is vital in alleviating protozoan parasite infections (**Figure 8**). Therefore, by targeting caspase-3 or GSDME, it holds much hope to explore the mechanism of development and treatment in inflammatory disorders, and to search new targets and strategies that are feasible for clinical use.

Recent studies have significantly advanced our understanding of the mechanisms and physiological roles of pyroptosis. Overall, there is a remarkable knowledge gap between GSDMs and protozoan parasite infection. More in-depth research needs to be focused on delineating how diverse immune cells regulate GSDMs pore-forming activity differently, how parasites manipulate GSDMs pore-forming activity to prevent being eradicated, what determines whether GSDMD cleavage induces hyperactivation or pyroptosis, how GSDMs pores initiate membrane repair, and what are the differences between extracellular and intracellular parasites in triggering GSDMs pore formation. Given the primary function of GSDMs in combating protozoan parasites, the search for GSDM-regulating pore-forming activity is likely to be extraordinarily developed in the future.

## Author Contributions

KC and SW contributed and design the content of the review. SW wrote the first draft of the manuscript and KC and FM edited the text. SW and FM did the recent published study that led to this review. All authors contribute to manuscript revision, read, and approved the submitted version.

## Funding

This work was funded by a Discovery Grant (RGPIN/04139-2019) from the Natural Sciences and Engineering Research Council of Canada and project grants from the Canadian Institutes of Health Research (PJT-173551, PJT-162284) awarded to KC.

## Conflict of Interest

The authors declare that the research was conducted in the absence of any commercial or financial relationships that could be construed as a potential conflict of interest.

## Publisher’s Note

All claims expressed in this article are solely those of the authors and do not necessarily represent those of their affiliated organizations, or those of the publisher, the editors and the reviewers. Any product that may be evaluated in this article, or claim that may be made by its manufacturer, is not guaranteed or endorsed by the publisher.
